# Chronotypes in Patients with Epilepsy: Does the Type of Epilepsy Make a Difference?

**DOI:** 10.1155/2015/941354

**Published:** 2015-05-21

**Authors:** Hallie Kendis, Kelly Baron, Stephan U. Schuele, Bhavita Patel, Hrayr Attarian

**Affiliations:** Northwestern University Feinberg School of Medicine, 710 N Lake Shore Drive, Suite 1111, Chicago, IL 60611, USA

## Abstract

Circadian rhythms govern all biological functions. Circadian misalignment has a major impact on health. Late chronotype is a risk factor for circadian misalignment which in turn can affect the control of seizures in epilepsy patients. We compared a group of 87 confirmed epilepsy patients regardless of subtypes with age- and sex-matched healthy controls. We compared generalized epilepsy patients with localization related epilepsy patients and with healthy controls. We found that primary generalized epilepsy patients were 5 times more likely to have a late chronotype than healthy controls. We did not find any significant differences between localization related epilepsy patients and healthy controls or between the overall epilepsy cohort and healthy controls. Generalized epilepsy patients are more likely to be evening types as compared to those with focal epilepsy or subjects without epilepsy. Epilepsy patients do not experience the same age related increase in morningness as do age-matched healthy controls. This is important in regard to timing of AED, identifying and preventing sleep deprivation, and integrating chronotype evaluations and chronotherapy in comprehensive epilepsy care. Further studies, using objective phase markers or the impact of chronotherapy on seizure control, are necessary.

## 1. Introduction

The circadian rhythm is the internal daily rhythm of all biological functions and particularly that of sleep/wake cycle. Circadian misalignment has a major impact on health and on the pathophysiology and therapeutics of many illnesses [1]. Individuals with a late chronotype are at risk for circadian misalignment because a chronotype is an individual's preference for daytime versus nighttime activity [2]. Larks, or morning-type individuals, wake up early in the morning, go to bed early in the evening, and have more energy and are most productive earlier in the day. In contrast, owls, or evening types sleep in later in the morning, stay up late into the night or early morning hours, and find the most productive hours to be later in the day.

Some evidence suggests that chronotype may play a role in epilepsy therapeutics. The most common reason for breakthrough seizures leading to hospitalization is subtherapeutic antiepileptic (AED) levels presumably due to missed doses [3]. Many patients do not even remember skipping a dose [3]. An epilepsy patient's chronotype has been shown to influence the timing of his medication [4] intake and its pharmacokinetics [5] both impacting AED levels. People with extremes of chronotypes, particularly those who are evening types, tend to be chronically sleep-deprived [6] which may also interfere with seizure control. Lastly, circadian misalignment may create a proinflammatory environment that may be more conducive to breakthrough seizures [7].

In 1957 Janz and Christian were the first to observe that patients with juvenile myoclonic epilepsy (JME), a type of primary generalized epilepsy, were more likely to fall asleep later and get up later than their peers and suffer from prolonged morning drowsiness and are most active in the afternoon and evening time [8]. In 2006, Pung and Schmitz matched 20 JME patients with 20 temporal lobe epilepsy (TLE) sufferers. Utilizing the Morningness-Eveningness Questionnaire (MEQ) [9], they discovered that those with JME had significantly lower scores (more evening type) than patients with TLE [10]. In contrast, Hofstra et al., in 2010, not only found no significant difference in MEQ scores between various types of epilepsy in a cohort of 200. They also noted that patients with epilepsy were, as a group, more morning types, than the general population [11]. The goal of our study was to elucidate the relationship between chronotype and the two main subtypes of epilepsy, localization related epilepsy versus primary generalized epilepsy, without focusing on one specific disorder or syndrome. Until now most studies have focused on JME patients and a few nocturnal frontal lobe epilepsy (TLE) patients. Many other patients thus have been excluded as none has looked at epilepsy patients as an inclusive group.

## 2. Methods

### 2.1. Participants

The Northwestern University IRB approved the study. Participants provided written informed consent to the study and received no compensation. The active group was recruited from the Northwestern Epilepsy Program Clinic. After a thorough review of their charts, patients who did not have definite diagnosis of epilepsy or who had an unknown subtype were excluded. Age- and sex-matched healthy controls were recruited from the employees and students of the Northwestern University's Downtown campus.

### 2.2. Procedure

All subjects filled out a Morningness-Eveningness Questionnaire (MEQ) and answered basic demographic questions as to age and sex and information about their seizure type, number of medications, and intractability of epilepsy was collected from their charts. The controls also filled out MEQs, questionnaires on their health history and provided basic demographic data.

### 2.3. Measures

Chronotype was determined by the MEQ. The questionnaire consists of 19 items regarding preference for sleep and wake times as well as the time of day the respondent feels at peak performance. There is a designated point value for each answer choice. Once totaled, there are 5 categories or chronotypes. The highest point values correlate with a definite morning type and the lowest point values correlate with a definite evening type. The questionnaire was externally validated by using 24-hour oral temperature curves [9]. We combined the 5 chronotypes used in the MEQ questionnaire into 3 categories. The definite and moderate morning types were combined in the morning-type category, while the definite and moderate evening types were combined into the evening-type category.

### 2.4. Statistical Analysis

We used Pair-matched case control study model. We used Fisher's exact and mid *P* exact tests to evaluate difference between groups. We also used the Mann-Whitney *U* test to compare medians between the groups.

## 3. Results

### 3.1. Epilepsy versus Age-Matched Controls

Demographic characteristics are listed in Table 1. The epilepsy group was comprised of 87 (32 men and 55 women) consecutive patients who were seen between January and April, 2013, at the Northwestern Epilepsy Program. The age range was from 18 to 83 years. Seventeen were diagnosed with idiopathic or cryptogenic generalized epilepsy and 70 had localization related epilepsy.

The control participants included 32 men and 55 women as well, who did not have any underlying neurologic or sleep disorders based on self-report and were between ages of 18–84 years.

There were no significant differences between the baseline ages or gender distribution in the epilepsy patients compared to the healthy controls. Mean age was 41.07 in the active group and 40.91 in controls, (*p* = 0.92). Mean age for focal epilepsy patients was 41 years and mean age for generalized epilepsy was 43 years (SD). Out of those with generalized epilepsy, 76% of patients were full-time students or were employed full-time, compared to 50% of patients with focal epilepsy.

Of all the patients with epilepsy, about 16% were evening types, 47% were intermediate types, and 37% were morning types. The mean MEQ score was not significantly different between the healthy controls (56.04, SD ± 10.1) versus those with epilepsy (53.4, SD ± 9.9) (*p* = 0.81). Although a higher percentage of patients with epilepsy were evening types (16% compared to 8% in healthy controls), the difference was not significant (*p* = 0.11).

When we broke down the cohort into a younger (18–49 years old) and older (≥50 years old) individuals, we discovered that older healthy controls had significantly higher mean MEQ scores (62.6, SD ± 7.5) compared to older epilepsy patients (55.5, SD ± 9.0) with a *p* value of 0.002. The same was not true for younger adults. Mean MEQ score for younger controls was 52.9, SD ± 9.7, and for younger epilepsy subjects it was 52.5 ± 10.3 with a *p* value of 0.83.

### 3.2. Focal or Localization Related Epilepsy versus Generalized Epilepsy

A comparison between those with focal epilepsy and those with generalized epilepsy revealed no significant difference in the mean MEQ score (55 versus 47, *p* = 0.31). A significant difference in the percentage of evening types between the two epilepsy groups was, however, seen. Eleven percent of patients with focal epilepsy were evening types compared to 36% of those with generalized epilepsy (*p* = 0.01). Out of those with generalized epilepsy, 76% of patients were full-time students or were employed full-time, compared to 50% of patients with focal epilepsy.

### 3.3. Healthy Controls versus Generalized Epilepsy

Only 8% of healthy controls were evening types versus 36% of those with generalized epilepsy. The odds ratio (OR) of being an evening type with generalized epilepsy compared to healthy controls was 5.05 (CI = 1.3–19.5, *p* = 0.019). In a binary logistic model predicting epilepsy diagnosis, after controlling for age and sex, epilepsy was still significant (*p* = 0.001).

## 4. Discussion

Our study found that prevalence of evening types was higher in the cohort of generalized epilepsy compared to both healthy controls and those with focal epilepsy. These findings were unrelated to employment or age. When we broke down the cohort into younger adults (ages 18–49) and older adults (≥50 years of age), we discovered that those with epilepsy did not experience the normal, age-related increase in morningness. Epilepsy, therefore, was associated with the dampening of the drive towards a more advanced circadian sleep wake rhythm.

The number of generalized epilepsy patients in our cohort was low, so overall there was no significant difference in mean MEQ scores or the proportion of evening types between the overall epilepsy cohort and age- and sex-matched controls.

The previous studies used JME patients as representatives of generalized epilepsy and either temporal lobe epilepsy patients as representatives of focal epilepsy or differentiated between temporal lobe and frontal lobe epilepsy patients. In our study we lumped all generalized epilepsy patients together and all focal epilepsy patients together. Janz and Christian's observations in the high prevalence of eveningness in JME patients did not control age or utilize a standardized chronotype questionnaire, as none were available then. Pung and Schmitz only compared patients with JME to patients with TLE. The fact that they only used TLE patients in the focal epilepsy group could have accounted for the significant difference between MEQ scores between the generalized and focal groups. TLE patients tend to have the highest MEQ scores; in Pung and Schmitz paper their mean score was 57 compared to 55 in our focal epilepsy group. Even so the difference of 10 points between JME and TLE in Pung and Schmitz was barely significant with a *p* value of 0.02. Hofstra's 2010 paper also found a MEQ score of 55 in TLE patients, 52 in FLE patients, and 53 in JME patients. The fact that Hofstra's JME patients had a higher score than our generalized epilepsy ones could be related to our sample's heterogeneous mix of generalized epilepsy patients. Lastly Hofstra's controls had a significantly lower mean MEQ (48.2) than ours (56.4). This may be due to geographic and cultural variations as well as time of the year when the data was collected [12]. We collected our data in spring and summer in central USA, while Hofstra collected their data continuously over 2 years in Northern Europe.

**Figure 1:**
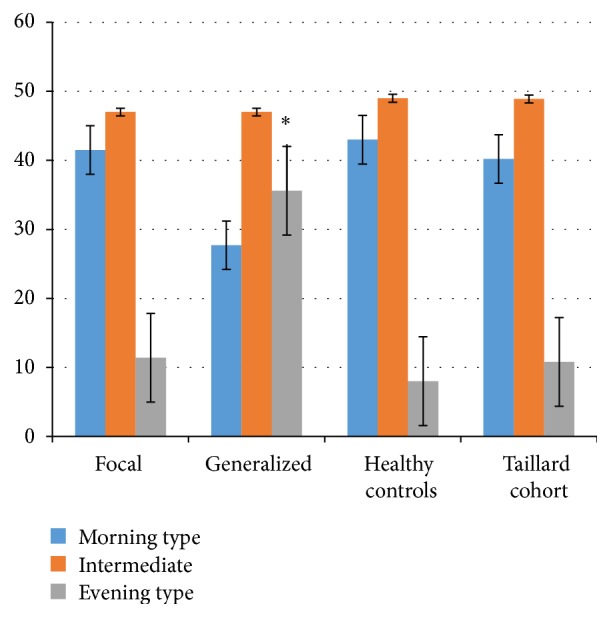
Distribution of different chronotypes among patients with generalized epilepsy, patients with focal epilepsy, healthy controls, and historic controls. The *Y*-axis indicated the percent of subjects in each chronotype as designated by the colored bar. ^*∗*^ *p* < 0.01.

Because of this we compared the distribution of chronotypes in our healthy controls to that of Taillard et al. Our results were very similar to those of their 2007 subjects with an age range of 18–81 [13] (see Figure 1).

The further understanding of the relationship between epilepsy types and individual chronotype or circadian rhythm will possibly impact and improve treatment. At a simplistic level, individuals who are more morning-type individuals versus individuals who are more evening-type individuals may alter the time they take their morning or nighttime antiepileptic medications. Some of these medications are very time sensitive and alterations in timing of dosing day-to-day, especially between workdays and free days, may impact serum levels and therefore seizure control. Hofstra et al. observed that evening-type individuals with epilepsy took their morning dose of medication on average 90 minutes later on free days than workdays [4]. In addition, sleep deprivation may be common in patients with epilepsy who are evening-type patients due to early morning work responsibilities and may reduce seizure control. Medication pharmacokinetics follows a circadian pattern and hence the blood levels of drugs may fluctuate based on when they are taken [5]. This may also impact the efficacy of AEDs. Lastly circadian misalignment can lead to a proinflammatory state, which may also worsen seizure control [14, 15]. All this work makes the evaluation of circadian misalignment and its treatment with chronotherapy an essential part of epilepsy care.

Our study, as well as those we referenced above, is limited because subjective questionnaires were used to evaluate chronotypes. Objective measures such as dim light melatonin onset (DLMO) through salivary or serum melatonin sampling [16], core body temperature curves, or actigraphic sleep wake recordings can more accurately and objectively determine the circadian phase. We did not evaluate and factor in the degree of seizure control in our epilepsy cohort. Our cohort was made up of a larger percentage of females as compared to males, which was matched with our control group as well, though sex can influence chronotype and both our cohort group and control group may be affected by the female predominance. We did not make the item of level data available as most of the epilepsy MEQ scales were collected as part of clinical evaluation and only the total score was reported. We, therefore, could not run our own internal validity analysis. Previous studies, however, have reported adequate internal consistency and factor validity [17]. Lastly, because we were limited to a single epilepsy center, we had a very small cohort of generalized epilepsy patients reflective of their proportion in the clinic population.

## 5. Conclusion

This study found that patients with generalized epilepsy are more likely to be evening-type patients as compared to those with focal epilepsy or subjects without epilepsy. In addition, older patients with epilepsy, regardless of type, do not exhibit the advancement in their sleep wake cycles that their healthy counterparts do. This not only may be an important consideration in regard to timing of AED, identifying and preventing sleep deprivation; this may also raise the possibility of integrating chronotype evaluations and chronotherapy in the comprehensive care of epilepsy patients.

Further studies using objective measures of circadian phase and further studies on the impact of chronotherapy on seizure control are necessary.

## Figures and Tables

**Table 1:** Distribution of epilepsy patients and health controls by gender and age.

	Generalized epilepsy Pts (*n* = )	Focal epilepsy Pts	Total epilepsy Pts	Healthy controls
% Female	76%	60%	63%	63%
Age 18–29	9	12	21	21
Age 30–39	4	26	30	30
Age 40–49	1	7	8	8
Age 50–59	1	15	16	16
Age 60–69	1	8	9	9
Age 70–79	1	0	1	1
Age 80–89	0	2	2	2
Totals	17	70	87	87
